# Supplementary material for: Transcriptional control of subtype switching ensures adaptation and growth of pancreatic cancer
Source: eLife. 2019 May 28;8:e45313. doi: 10.7554/eLife.45313 (PMC6538376; doi:10.7554/eLife.45313)
Supplement: Supplementary file 1. [file elife-45313-supp1.docx]

**Supplementary File 1**

Human and mouse primer sequences used in the study.

| **Human Primers** |  |  |
| --- | --- | --- |
| **Gene** | **FWD (5' to 3')** | **REV (5' to 3')** |
| 18S | GCTTGCGTTGATTAAGTCCC | GCCTCACTAAACCATCCAATC |
| GLI2 | TCAAGGAAGATCTGGACAGG | TGTGCTCGTTGTTGATGTG |
| GLI1 | AGCGTGAGCCTGAATCTGTG | CAGCATGTACTGGGCTTTGAA |
| SHH | CTACGAGTCCAAGGCACATATC | CAGGTCCTTCACCAGCTTG |
| SPP1 | CTCCATTGACTCGAACGACTC | CAGGTCTGCGAAACTTCTTAGAT |
| CD44 | CTGCCGCTTTGCAGGTGTA | CATTGTGGGCAAGGTGCTATT |
| VIM | AGTCCACTGAGTACCGGAGAC | CATTTCACGCATCTGGCGTTC |
| ZEB1 | GATGATGAATGCGAGTCAGATGC | ACAGCAGTGTCTTGTTGTTGT |
| ZEB2 | ATGACCTGCCACCTGGAACTC | GCGGTACTTGATGTGCTCCTTC |
| SNAI1 | TCGGAAGCCTAACTACAGCGA | AGATGAGCATTGGCAGCGAG |
| CDH2 | TCAGGCGTCTGTAGAGGCTT | ATGCACATCCTTCGATAAGACTG |
| CDH1 | ATTTTTCCCTCGACACCCGAT | TCCCAGGCGTAGACCAAGA |
| ESRP1 | ACTAAAATAGACGTCGAAAGCC | GCCCATCAGTACAGAGACAG |
| ESRP2 | ATGCAGCATCCAAGCACCT | CTGTCTCCAGTCCTAAACCCT |
| SOX2 | GCCGAGTGGAAACTTTTGTCG | GGCAGCGTGTACTTATCCTTCT |
| CD24 | CGCGGTCGCACTGGAAT | AAAGAAAAGTCCGCGCCTC |
| KRAS | ACAGAGAGTGGAGGATGCTTT | TTTCACACAGCCAGGAGTCTT |
| WNT7A | CTGTGGCTGCGACAAAGAGAA | GCCGTGGCACTTACATTCC |
| GATA6 | CTCAGTTCCTACGCTTCGCAT | GTCGAGGTCAGTGAACAGCA |
|  |  |  |
| Classical genes: |  |  |
| ATP10B | AAGAGCAGACCTATGTGCAGA | GCATATCCCATTGGGGTCAGAG |
| ST6GALNAC1 | AGAAAGGTCTCTACAGTCCCTG | TGTGTGTTGAGGGCATTGTTC |
| CAPN8 | CTCAAGGCATCATCTGGAAGC | ACCCTGACAAATGTCTGTGCG |
| CEACAM6 | TCAATGGGACGTTCCAGCAAT | CACTCCAATCGTGATGCCGA |
| CEACAM5 | AAGAAATGACGCAAGAGCCTATG | CCCGAAAGGTAAGACGAGTCTG |
| TFF1 | CCCCGTGAAAGACAGAATTGT | GGTGTCGTCGAAACAGCAG |
| AGR2 | GTCAGCATTCTTGCTCCTTGT | GGGTCGAGAGTCCTTTGTGTC |
| S100P | AAGGATGCCGTGGATAAATTGC | ACACGATGAACTCACTGAAGTC |
| SDR16C5 | TATACCTGCGATTGCAGCCAA | CGATTCCGGCATTGTTGATTAGG |
| GPX2 | GAATGGGCAGAACGAGCATC | CCGGCCCTATGAGGAACTTC |
| ELF3 | GGCCGATGACTTGGTACTGAC | GCTTGCGTCGTACTTGTTCTTC |
| ERBB3 | GACCCAGGTCTACGATGGGAA | GTGAGCTGAGTCAAGCGGAG |
| TMEM45B | GCTTCCAGGGAGTTTCTTCCT | CTTCCGCGTGTGGCTAAAGTA |
| TOX3 | ATTCCACCAATCACGCCTCC | GGATCGCTGAGGGCTTGAAA |
| TSPAN8 | ACTTCTTGTTCTGGCTATGTGG | CACAGCAACGTAGGAGCTAGA |
| FXYD3 | GGCTTAAGAGGCCCGAGTTT | TGCATTTTGCACTCATGACGA |
| FOXQ1 | CACGCAGCAAGCCATATACG | CGTTGAGCGAAAGGTTGTGG |
| LGALS4 | CGACGCTGCCTTACTACCAG | CCAACCACAAAGTTCACGAAGA |
| PLS1 | ACAAGAGGGAAGGGATTACTGC | AGATGCTTACAGTCAGGGTCATT |
|  |  |  |
| Basal-like genes: |  |  |
| LY6D | CCAGCAACTGCAAGCATTC | CACAGTCCTTCTTCACCAGATT |
| LEMD1 | ATTGCAGAACCAACTTGAGAAGC | CGCGCAGTAGTCTCTCTCTT |
| KRT15 | TCTGCTAGGTTTGTCTCTTCAGG | CCAGGGCACGTACCTTGTC |
| CTSL2 | CGTGACGCCAGTGAAGAATCA | CGCTCAGTGAGACAAGTTTCC |
| DHRS9 | CTGTGGACTCGTAAAGGAAAACT | GCAGCGATTACATGAAATCCCT |
| AREG | GAGCCGACTATGACTACTCAGA | TCACTTTCCGTCTTGTTTTGGG |
| CST6 | TACTTCCTGACGATGGAGATGG | GAGTTCTGCCAGGGAACCAC |
| SERPINB4 | CTGGGTGGAAAGTCAAACGAA | TGTCGTATCATTGCCAATAGTCC |
| SERPINB3 | CGCGGTCTCGTGCTATCTG | ATCCGAATCCTACTACAGCGG |
| S100A2 | ATGAGTGGGAATGGCAAGAG | CTCCCAGGGTGAGGATTTATATG |
| FGFBP1 | GGAAACAAGTTGCCCGGAATC | AATAGAGTGGAGCTGACTAGCTT |
| SPRR3 | CCAGCAGAAGCAGACCTTTAC | TCCTTGGTTGTGGGAACAAATA |
| SPRR1B | TCCCCTATCCCATTCTGCGT | AGCAGCTGAAAACTAGCTCTGG |
| UCA1 | GCCAGCCTCAGCTTAATCCA | CCCTGTTGCTAAGCCGATGA |
| KRT14 | GTGGGTGGAGATGTCAATGT | CATCCTTGCGGTTCTTCTCT |
| KRT6A | TCTCACTGTTGGTAAAGCCCAG | CTGGCTGAGTTGGCACTGAA |
| KRT6C | GGGTTTCAGTGCCAACTCAG | CCAGGCCATATAAGCTGCGG |
| PAPPA | ACAAAGACCCACGCTACTTTTT | CATGAACTGCCCATCATAGGTG |
| HMMR | AGAACCAACTCAAGCAACAGG | AGGAGACGCCACTTGTTAATTTC |
| CKS2 | TTCGACGAACACTACGAGTACC | GGACACCAAGTCTCCTCCAC |
| FERMT1 | GGTGAGGTTGCGAGTCAGC | CCAGACGGCTTTAACAAGGAA |
| TWIST1 | GTCCGCAGTCTTACGAGGAG | GCTTGAGGGTCTGAATCTTGCT |
| FAM83A | GGAGATGTGTGACAAAGTCCAG | CCAGCGAATTTCCTGCCTG |
| KRT17 | GCCGCATCCTCAACGAGAT | CGCGGTTCAGTTCCTCTGTC |
| SCEL | TCGGTACAGTTCTGATGACACT | AACATGGACATGCTCCTATTGG |
| KRT7 | CATGCAGGATGTGGTGGAGG | CCGTCTCATTGAGGGTCCTG |
| GPR87 | GCCAGGAAAGAACACCACCC | GATCCACACTGCTAAACCATTCA |
| SLC2A1 | ATTGGCTCCGGTATCGTCAAC | GCTCAGATAGGACATCCAGGGTA |
| ANXA8L2 | AAAGCCATGAAGGGGATCGG | GTTACAGCACAAGTGACCCTG |
| TNS4 | AGCCAGGGGCTTTTGTCATAA | AGACGACTCGATGAGGAAGTG |
| VGLL1 | TCAGAGTGAAGGTGTGATGCT | GCACGGTTTGTGACAGGTACT |
|  |  |  |
| **Mouse primers** |  |  |
| 18S | GTAACCCGTTGAACCCCATT | CCATCCAATCGGTAGTAGCG |
| Gli2 | CAACGCCTACTCTCCCAGAC | GAGCCTTGATGTACTGTACCAC |
| Gli1 | CTACTCGGGGTTCAATGATGC | TGTGGAGTTGGGGCTAGACAT |
| Vim | CGTCCACACGCACCTACAG | GGGGGATGAGGAATAGAGGCT |
| Zeb1 | ACCGCCGTCATTTATCCTGAG | CATCTGGTGTTCCGTTTTCATCA |
| Kras | CAAGAGCGCCTTGACGATACA | CCAAGAGACAGGTTTCTCCATC |
|  |  |  |
| Basal-like genes: |  |  |
| Ly6d | GCCTGGGCACTTCGATGTC | TGAGTTTGCACACTCTTTCCTC |
| Lemd1 | GACTATGAGTTGCACAAGCATCT | TCTTCTCGTAGGTCTTTCTGGTG |
| Dhrs9 | ATGCTGTTTTGGTTGTTGGCT | GTTCTGGCTGCTAAGTTTCCA |
| Cst6 | GAACTTGTCACCCACCGACC | TTTGGTGTCTCGGAAGTAGTAGA |
| S100a2 | ACGCCAGTCAAGAGGACGA | CCCCACATAGCTCAGCAGC |
| Fgfbp1 | TGGCTACTCAGGCGTTCTCA | CGTCAGAGATTTAGATGTCCTGC |
| Sprr3 | GAACAGCATCAAGTGAAGCAAC | CTGGAATCTTGGTGTTTCCTGG |
| Krt14 | AGCGGCAAGAGTGAGATTTCT | CCTCCAGGTTATTCTCCAGGG |
| Pappa | GGATGGGTCATGGGCATTCA | GAAAAAGTAGCGTGGATCTCTGT |
| Cks2 | TCGATGAGCACTACGAGTACC | CCATCCTAGACTCTGTTGGACAC |
| Twist1 | GGACAAGCTGAGCAAGATTCA | CGGAGAAGGCGTAGCTGAG |
| Fam83a | ATGAGTCGGTCAAGGCATGTG | TGAGGACAGGAAGTCTACCTCT |
| Scel | GACAACAGGGTTTTCAGGACG | TACCGGCTAATTGTGGCTTTT |
| Vgll1 | TGTCTGGATACCTGAAAGCAGT | GGCCTCTTGAGGTTACGCA |
| Gpm6b | CAGGCACCGTGGCAATTCT | GTTGGATCACTTCACTCAGCAA |
| Slc16a1 | GAGGTGGAGCTGACGAGGT | CATGGACACGAAGAGCACCC |
| Aim2 | GTCACCAGTTCCTCAGTTGTG | CACCTCCATTGTCCCTGTTTTAT |
|  |  |  |
| **shRNA sequences:** |  |  |
| HUMAN: |  |  |
| KRAS | TRCN0000010369 | CAGTTGAGACCTTCTAATTGG |
| KRAS | TRCN0000033263 | GACGAATATGATCCAACAATA |
| GLI2 | TRCN0000033329 | CCGCTTCAGATGACAGATGTT |
| GLI2 | TRCN0000033332 | GCTCTACTACTACGGCCAGAT |
|  |  |  |
|  |  |  |
| MOUSE: |  |  |
| Gli2 | TRCN0000226034 | TCGACCTACAACGCATGATTCC |
| Gli2 | TRCN0000226035 | TGTGGAGGACTGCCTACATAT |
| Gli2 | TRCN0000219066 | TATGTTTACCCGCTCCTATTT |
|  |  |  |
