# Supplementary material for: Transcriptional control of subtype switching ensures adaptation and growth of pancreatic cancer
Source: eLife. 2019 May 28;8:e45313. doi: 10.7554/eLife.45313 (PMC6538376; doi:10.7554/eLife.45313)
Supplement: Supplementary file 2. — List of genes associated with the classical and basal-like gene signatures and their expression in the corresponding Moffitt, Collisson and Bailey signatures. [file elife-45313-supp2.docx]

**Supplementary File 2**

**Classical and basal-like gene signatures:** List of genes associated with the classical and basal-like gene signatures and their expression in the corresponding Moffitt, Collisson, and Bailey signatures

| Gene | Moffitt | Collisson | Bailey |
| --- | --- | --- | --- |
| ATP10B |  | X | X |
| ST6GALNAC1 | X | X | X |
| CAPN8 |  | X | X |
| CEACAM6 | X | X | X |
| CEACAM5 |  | X |  |
| TFF1 | X | X | X |
| AGR2 | X | X | X |
| S100P |  | X |  |
| SDR16C5 |  | X |  |
| GPX2 |  | X | X |
| ELF3 |  | X |  |
| ERBB3 |  | X | X |
| TMEM45B |  | X |  |
| TOX3 |  | X | X |
| TSPAN8 | X | X | X |
| FXYD3 |  | X |  |
| FOXQ1 |  | X |  |
| LGALS4 | X | X | X |
| PLS1 |  | X | X |
|  |  |  |  |
| **Basal-like signature genes** |  |  |  |
| Gene | Moffitt | Collisson | Bailey |
| LY6D | X |  | X |
| LEMD1 | X |  |  |
| KRT15 | X |  |  |
| CTSL2 | X |  |  |
| DHRS9 | X |  |  |
| AREG | X |  |  |
| CST6 | X |  | X |
| SERPINB4 | X |  |  |
| SERPINB3 | X |  |  |
| S100A2 | X | X | X |
| FGFBP1 | X |  |  |
| SPRR3 | X |  | X |
| SPRR1B | X |  | X |
| UCA1 | X |  |  |
| KRT14 |  | X | X |
| KRT6A | X |  | X |
| KRT6C | X |  | X |
| PAPPA |  | X |  |
| HMMR |  | X |  |
| CKS2 |  | X |  |
| FERMT1 |  | X |  |
| TWIST1 |  | X | X |
| FAM83A | X |  | X |
| KRT17 | X |  | X |
| SCEL | X |  |  |
| KRT7 | X |  |  |
| GPR87 | X |  | X |
| SLC2A1 | X |  |  |
| ANXA8L2 | X |  |  |
| TNS4 | X |  | X |
|  |  |  |  |
